# Structural and social changes due to the COVID-19 pandemic and their impact on engagement in substance use disorder treatment services: a qualitative study among people with a recent history of injection drug use in Baltimore, Maryland

**DOI:** 10.1186/s12954-024-01008-8

**Published:** 2024-05-08

**Authors:** Eshan U. Patel, Suzanne M. Grieb, Abigail K. Winiker, Jennifer Ching, Catherine G. Schluth, Shruti H. Mehta, Gregory D. Kirk, Becky L. Genberg

**Affiliations:** 1 Department of Epidemiology, Johns Hopkins Bloomberg School of Public Health, 615 N. Wolfe Street, Baltimore, MD 21205 USA; 2 Department of Pediatrics, Johns Hopkins University School of Medicine, Baltimore, MD USA; 3 Department of Health, Behavior and Society, Johns Hopkins Bloomberg School of Public Health, Baltimore, MD USA; 4 Department of Medicine, Johns Hopkins University School of Medicine, Baltimore, MD USA

**Keywords:** Opioid use disorder, Medication for opioid use disorder, Opioid agonist therapy, Recovery support, Fentanyl

## Abstract

**Background:**

Substance use disorder treatment and recovery support services are critical for achieving and maintaining recovery. There are limited data on how structural and social changes due to the COVID-19 pandemic impacted individual-level experiences with substance use disorder treatment-related services among community-based samples of people who inject drugs.

**Methods:**

People with a recent history of injection drug use who were enrolled in the community-based AIDS Linked to the IntraVenous Experience study in Baltimore, Maryland participated in a one-time, semi-structured interview between July 2021 and February 2022 about their experiences living through the COVID-19 pandemic (n = 28). An iterative inductive coding process was used to identify themes describing how structural and social changes due to the COVID-19 pandemic affected participants’ experiences with substance use disorder treatment-related services.

**Results:**

The median age of participants was 54 years (range = 24–73); 10 (36%) participants were female, 16 (57%) were non-Hispanic Black, and 8 (29%) were living with HIV. We identified several structural and social changes due the pandemic that acted as barriers and facilitators to individual-level engagement in treatment with medications for opioid use disorder (MOUD) and recovery support services (e.g., support group meetings). New take-home methadone flexibility policies temporarily facilitated engagement in MOUD treatment, but other pre-existing rigid policies and practices (e.g., zero-tolerance) were counteracting barriers. Changes in the illicit drug market were both a facilitator and barrier to MOUD treatment. Decreased availability and pandemic-related adaptations to in-person services were a barrier to recovery support services. While telehealth expansion facilitated engagement in recovery support group meetings for some participants, other participants faced digital and technological barriers. These changes in service provision also led to diminished perceived quality of both virtual and in-person recovery support group meetings. However, a facilitator of recovery support was increased accessibility of individual service providers (e.g., counselors and Sponsors).

**Conclusions:**

Structural and social changes across several socioecological levels created new barriers and facilitators of individual-level engagement in substance use disorder treatment-related services. Multilevel interventions are needed to improve access to and engagement in high-quality substance use disorder treatment and recovery support services among people who inject drugs.

**Supplementary Information:**

The online version contains supplementary material available at 10.1186/s12954-024-01008-8.

## Background

The United States (US) has experienced multiple intersecting public health crises, including the COVID-19 pandemic and the overdose epidemic. Between 2000 and 2018, there was a three-fold increase in overdose deaths and an eight-fold increase in injection-involved overdose deaths in the US [1], and the COVID-19 pandemic further accelerated overdose mortality rates [2]. People who inject drugs (PWID) are disproportionately at risk of additional health harms as compared to people who use drugs via other routes of administration. Consistent with nationwide increases in the number of PWID before the pandemic [3], there were increases in injection-related bacterial infections [4, 5], acute hepatitis C virus (HCV) infections [6, 7], and localized human immunodeficiency virus (HIV) infection outbreaks [8–11]. The impact of the COVID-19 pandemic on these injection-related harms remains elusive [12]. PWID have also faced a higher risk of SARS-CoV-2 infection as compared to the general population in some settings [13], and substance use disorders have been associated with an increased risk of COVID-19-related complications [14–17]. Pandemic-related changes in access to and engagement in substance use disorder treatment-related services among PWID may influence the trajectory of several public health crises [12, 18].

Substance use disorder treatment-related services, including medications for opioid use disorder (MOUD), are key to the prevention of drug-related harms. Although treatment with MOUD, particularly methadone and buprenorphine, reduces the risk of HIV and HCV acquisition [19, 20], improves HIV and HCV testing and treatment outcomes [21–24], and decreases the risk of overdose and all-cause mortality [25], less than 15% of people with opioid use disorder in the US used MOUD in 2019 [26]. Recovery support services—including self-help/mutual aid recovery support groups and peer recovery coaches—also aid people in achieving and maintaining recovery from substance use disorders [27]. Engagement in recovery support groups has been associated with improved substance use outcomes [28–31]. Peer recovery support services, which specifically foster social connections with persons of shared lived experience, have been shown to be effective at promoting MOUD initiation, enhancing engagement in recovery support groups, and improving substance use outcomes [32–35].

Historically, PWID have faced many structural barriers to substance use disorder treatment that constrain the context within which social barriers operate. For instance, poor access to and engagement in treatment with MOUD is shaped by structural stigma and discrimination including through rigid regulatory policies (e.g., near-daily in-person visits to federally-certified Opioid Treatment Programs [OTPs] for methadone treatment and low thresholds for expulsion) [36–38]. Social factors that impede engagement in substance use disorder treatment services include unstable housing [39, 40], poverty [39, 41], incarceration [42, 43], lack of social support [44, 45], and various other manifestations of social stigma [46, 47]. These structural and social factors also influence individual-level psychosocial barriers to engagement in substance use disorder treatment, such as ongoing substance use, negative emotional responses, and negative mental health symptoms [48]. Thus, even before the COVID-19 pandemic, there were a multitude of multilevel barriers to engagement in substance use disorder treatment services, especially among PWID.

There have been many structural and social changes due to the COVID-19 pandemic. Notably, substance use disorder treatment provision and the regulatory environment markedly changed in response to the COVID-19 public health emergency. In the US, there were many regulatory policy changes regarding MOUD provision to ease access and minimize in-person encounters [49–52]. On March 16, 2020, the Drug Enforcement Administration (DEA) permitted controlled substances, including buprenorphine, to be prescribed by telemedicine (i.e., via an “audio-visual, real-time, two-way interactive communication system”) even for *new* patients without a prior in-person evaluation visit. This policy was extended to allow audio-only communication systems on March 31, 2020. In partnership with the DEA, the Substance Abuse and Mental Health Services Administration (SAMHSA) also issued guidance on March 16, 2020 to State Opioid Treatment Authorities that they could allow OTPs to request flexibility for “take-home” methadone doses; “stable” patients could receive up to 28 days of methadone doses and “less stable” patients could receive up to 14 days of methadone doses. Telehealth was also allowed to be used to conduct the counseling required for methadone administration and dispensation. Many substance use disorder treatment programs adapted by providing take-home methadone doses and leveraging telehealth for buprenorphine prescribing and delivering behavioral health counseling and other recovery support services during the pandemic; however, some programs closed altogether while others suspended some services and faced other operational and logistical barriers (e.g., staff shortages and limited in-person capacity) [49–51, 53–58].

Other structural changes associated with the onset of the COVID-19 pandemic include increased awareness of harm reduction principles in the context of the pandemic [59], strained healthcare systems [60, 61], decreased access to general health services [62, 63], changes in public transportation systems [64], global supply chain interruptions [65], and changes to the illicit drug market with reduced availability of illicit drugs in some settings [66, 67]. In addition to these structural changes, there have been many social consequences of the COVID-19 pandemic for PWID, such as resource loss including financial resources and increased social isolation [66, 68, 69]. Many of these structural and social consequences of the COVID-19 pandemic have also been reported by people who use drugs, including PWID, globally [53, 70–74], highlighting the need to understand the downstream health-related impacts of these changes. Indeed, the dynamic structural and social context of the COVID-19 pandemic has been linked to increased negative emotional and mental health symptoms among PWID [62, 66, 68, 69]. Furthermore, the COVID-19 pandemic has been associated with changes in individual-level behaviors, including substance use and injection practices [67, 75]. For example, PWID in Baltimore increasingly transitioned from injection to non-injection drug use during the pandemic [75], and there is also evidence of increased solitary drug use [76].

In accordance with the socioecological model [77]—which considers the ways in which multi-level external forces interact to influence individual-level health behaviors and outcomes—the structural and social changes due to the COVID-19 pandemic at multiple socioecological levels may have also impacted individual-level engagement in substance use disorder treatment and related services [12]. While many quantitative studies have reported transient disruptions in substance use disorder service provision because of the pandemic, there is evidence that the regulatory changes for MOUD generally either sustained pre-pandemic access or improved access to MOUD during the pandemic among people with opioid use disorder in the US [50, 51, 78]. However, variation in findings exist across populations. One study of PWID in New York City found that the pandemic was associated with reductions in buprenorphine use and no significant change in methadone use [68]. Even in settings outside the US, there have been reports of both positive and negative effects of the pandemic on the population-level prevalence of MOUD use among PWID [79–81]. Exploring the disruptions, or lack thereof, in access to and use of substance use treatment-related services during the pandemic can help illuminate more nuanced factors influencing the varied findings from these quantitative studies.

There are limited qualitative studies, however, characterizing the impact of the COVID-19 pandemic on substance use disorder treatment-related services among people with a history of injection drug use in the community who are not necessarily engaged in care. Two prior qualitative studies of pandemic-related impacts on substance use disorder treatment among PWID in the US were restricted to individuals engaged in harm reduction and other health services during the pandemic [62, 82], while one such qualitative study with community-based sampling was restricted to women without HIV who injected drugs in the past 6 months [83]. Given that substance use disorders are a chronic condition and PWID can transition in and out of injecting drug use over time [84, 85], it is important to characterize the experiences of people with a history of injection drug use who are and are not actively injecting drugs. Data on how the structural and social disruptions due to the pandemic influenced experiences with MOUD treatment and recovery support services from the perspective of people with a history of injection drug use in the community who may not have had access to these services during the pandemic are needed. These data can inform health policies and implementation practices that will optimize the delivery of substance use disorder treatment and related services during future social disruptions and may also provide important lessons applicable to health service delivery for this priority population in everyday practice.

The objective of this study was to qualitatively explore how structural and social changes due to the COVID-19 pandemic influenced individual-level experiences with substance use disorder treatment services among a community-based sample of people with a recent history of injection drug use in Baltimore, Maryland.

## Methods

### Study participants

This analysis used data from the Covid Health and Network Group Experience Study (CHANGES), which included data from 28 in-depth interviews conducted with people with a recent history of injection drug use in Baltimore, Maryland between July 2021 and February 2022. The overarching objective of the CHANGES study was to broadly explore the impact of the COVID-19 pandemic on the day-to-day lives, social networks, drug use, health-related behaviors, and health service use of people with a recent history of injection drug use in Baltimore. CHANGES participants were recruited from the AIDS Linked to IntraVenous Experience (ALIVE) study, an ongoing longitudinal cohort study of community-recruited people with a history of injection drug use living in or near Baltimore [86]. Individuals had to be 18 years of age and older to be eligible for enrollment in the ALIVE study. Notably, ALIVE study participants include individuals who are in and out of care for substance use disorder treatment [85]. For instance, approximately half the cohort reported being prescribed MOUD in the past 6 months at their first semi-annual visit between 2015 and 2019 [87].

In the first phase of data collection for the CHANGES study, we sampled ALIVE study participants who reported active injection drug use in the past 12 months during a drug network survey conducted in 2018–2019. A research assistant (A.K.W., C.G.S., or J.C.) re-contacted eligible participants via telephone and asked whether they were interested in participating in a qualitative sub-study. In the second phase of data collection for the CHANGES study, individuals who reported injection drug use and/or experiencing housing instability in the past 6 months during routine semi-annual ALIVE study visits were asked either in-person by ALIVE study staff or via telephone by a research assistant (J.C. or A.K.W.) whether they were interested in participating in a qualitative sub-study. Participants with ongoing injection drug use and those experiencing housing instability were intentionally oversampled in the second phase of data collection because of the iterative process of data collection (i.e., individuals actively injecting and experiencing housing instability were describing disproportionate impacts from the pandemic and we sought to follow this group until saturation was achieved) and because the ALIVE study returned to in-person modes of data collection during recruitment for the CHANGES study, thereby increasing the feasibility of recruiting participants with these characteristics. Interested individuals were read an informed consent document outlining the study’s goals and procedures. Individuals interested in participating in the study provided oral informed consent and scheduled a time to complete a one-time, telephone-based interview. Almost all individuals who were successfully contacted agreed to participate in the study. Individuals received $25 for their time and participation in the study. The study protocol was approved by the Johns Hopkins Bloomberg School of Public Health Institutional Review Board.

### Data collection

The CHANGES study team developed an open-ended, semi-structured interview guide that consisted of questions informed by the socioecological model and aimed to assess the ways in which the structural and social disruptions of the COVID-19 pandemic impacted the experiences of PWID at the individual-, interpersonal-, community-, and society-level [77]. For instance, questions prompted participants to identify pandemic-related changes in their experiences with housing, income, relationships, caretaker responsibilities, drug use practices, and healthcare access including drug treatment. The interview guide was pilot tested with the ALIVE study staff and is provided in Additional file 1.

The 28 in-depth interviews were conducted by two research assistants (A.K.W. and J.C.). At the start of each interview, the research assistants introduced themselves and the study’s goals and strove to establish rapport with each participant to maximize their comfort during the conversation. Neither interviewer had an established relationship with the study participants prior to study commencement. No personal characteristics were reported about the interviewer to participants. The study team met regularly to discuss emergent findings and critically evaluate potential biases and assess the ways in which the positionality of the research assistants could influence the study results throughout the data collection and analysis process.

The interviews lasted between 20 and 90 min. The interviewers wrote memos following each interview. Data collection proceeded until thematic saturation was achieved. To assess this, the study team met weekly to discuss new interviews and emergent findings, determining that inductive thematic saturation was reached when additional data no longer yielded new emergent codes or themes [88]. Codebook development began while data collection was ongoing, enabling the team to assess whether each subsequent interview contributed to new code development. All interviews were audio recorded, transcribed verbatim, and reviewed for accuracy by the research assistants (A.K.W. and J.C.). Transcripts were not returned to participants for comment and/or correction. Transcripts were de-identified and uploaded into Atlas.ti version 9 to facilitate data management and analysis [89].

### Data analysis

Interview data were analyzed using an iterative, thematic constant comparison process informed by grounded theory [90, 91].

As part of the CHANGES study, a research assistant (J.C.) and a senior qualitative researcher (S.M.G.) each read the first six interviews, independently creating an initial open-code list. These open-code lists were merged, and a preliminary codebook of the inductive codes, their definitions, and an exemplar text segment was drafted. This initial codebook was reviewed in detail with the second research assistant (A.K.W.). The research assistants then independently coded an additional three transcripts and met with the senior qualitative researcher to identify, discuss, and reconcile any discrepancies in their codebook application. The codebook was subsequently adapted as needed to improve definitional precision and clarity of themes/codes. This process continued iteratively, with the research assistants each continuing to independently code the same transcripts and discuss codebook application as a team, until a Krippendorff’s c-α-binary score ≥ 0.70 was achieved, indicating sufficient intercoder agreement. The research assistants then conducted split coding for the remaining transcripts with coding checks by the senior qualitative researcher.

As a sub-analysis of the CHANGES study, the present analysis focused on exploring how structural and social changes due to the COVID-19 pandemic affected experiences with substance use disorder treatment-related services. After listening to all 28 audio recordings and reading all transcripts and memos in their entirety, the lead investigator of this analysis (E.U.P.) reviewed all text segments tagged with substance use disorder treatment-related codes (i.e., “drug treatment relationships”, “MOUD perceptions”, “pre-COVID-19 drug treatment” and “drug treatment during COVID-19”). All 28 participants were represented in this sub-set of the data. The substance use disorder treatment-related text segments were iteratively compared within and between interviews. The data were further categorized based on emergent and salient themes after regular discussion and reflection with the entire research team, including the senior qualitative researcher (S.M.G.) who also reviewed all the substance use disorder treatment-related text segments. After identifying structural and social changes due to the COVID-19 pandemic, the data were interrogated to understand how they influenced individual-level engagement in substance use disorder treatment services. Emergent themes were ultimately interpreted and organized as barriers or facilitators to substance use disorder treatment-related service engagement in the context of the COVID-19 pandemic and were situated within the socioecological model. Organization of emergent themes using a barriers and facilitators framework within the socioecological model highlighted the meaning of the structural and social consequences of the COVID-19 pandemic in relation to substance use disorder treatment-related service utilization and allowed interpretation and presentation of the results using established constructs that can be effectively used to improve policy and implementation practices. We considered barriers as factors or processes that hindered an individuals’ engagement in substance use disorder treatment-related services and facilitators as factors or processes that enabled an individuals’ engagement in substance use disorder treatment-related services. Variability of the data was considered based on age, sex, and race. Participant checking was not performed; however, the qualitative data are presented using direct quotes from participants to illustrate the study findings. The COnsolidated criteria for Reporting Qualitative research (COREQ) checklist is provided in Additional file 2.

Descriptive statistics were used to describe characteristics of study participants using Stata/SE, version 17 (StataCorp LLC, College Station, TX). Demographic data were obtained from the most recent semi-annual study visit in the parent ALIVE study [86]; data on experiences with homelessness and engagement in substance use disorder-related treatment during the COVID-19 pandemic were ascertained from the interview.

### Researcher positionality

Both research assistants (A.K.W. and J.C.) who led the interviews and coding had formal training in public health and qualitative methods and had prior experience conducting in-depth interviews with priority populations. A.K.W. is a white female and at the time of the study was a PhD student in public health. J.C. is an Asian female who at the time of this study was a research program coordinator for the ALIVE study.

The post-coding analysis was led by an Asian Indian, male (E.U.P.) who at the time of this study was a PhD candidate in infectious disease epidemiology. This analysis contributed to his doctoral training in qualitative methods and his dissertation research on the impact of the COVID-19 pandemic on determinants of HIV and HCV transmission, risk, and prevention among PWID in Baltimore. He has been an active collaborator of the ALIVE study since 2014. E.U.P. had a family member engaged in substance use disorder treatment services during the COVID-19 pandemic.

Data collection and analyses were supervised by two white, female senior researchers—a qualitative researcher and anthropologist (S.M.G.) and a mixed-methods, health services researcher and epidemiologist (B.L.G.)—both of whom have over a decade of experience conducting qualitative research with priority populations. E.U.P., S.M.G., and B.L.G. did not interact with the study participants.

## Results

### Sample characteristics

Of the 28 participants, 10 (36%) participants were female, 16 (57%) identified as non-Hispanic Black, 11 (39%) identified as non-Hispanic white, and one (4%) participant reported being another unspecified non-Hispanic race. Participants’ median age was 54 years (range: 24–73). Half (50%) of the participants had attained less than a high school education, 6 (21%) participants completed high school or received their GED, and 8 (29%) participants had attained some college education or more. Eight (29%) participants were living with HIV. During the qualitative interviews, 10 (36%) participants reported experiencing homelessness at some point during the pandemic. In addition, 21 (75%) participants reported some form of engagement in substance use disorder treatment-related services at some point during the pandemic, including 17 (61%) participants who reported prescribed MOUD use during the pandemic. Prescribed buprenorphine use during the pandemic was infrequently reported (n = 2 [7%]).

### Overall findings

**Fig. 1:**
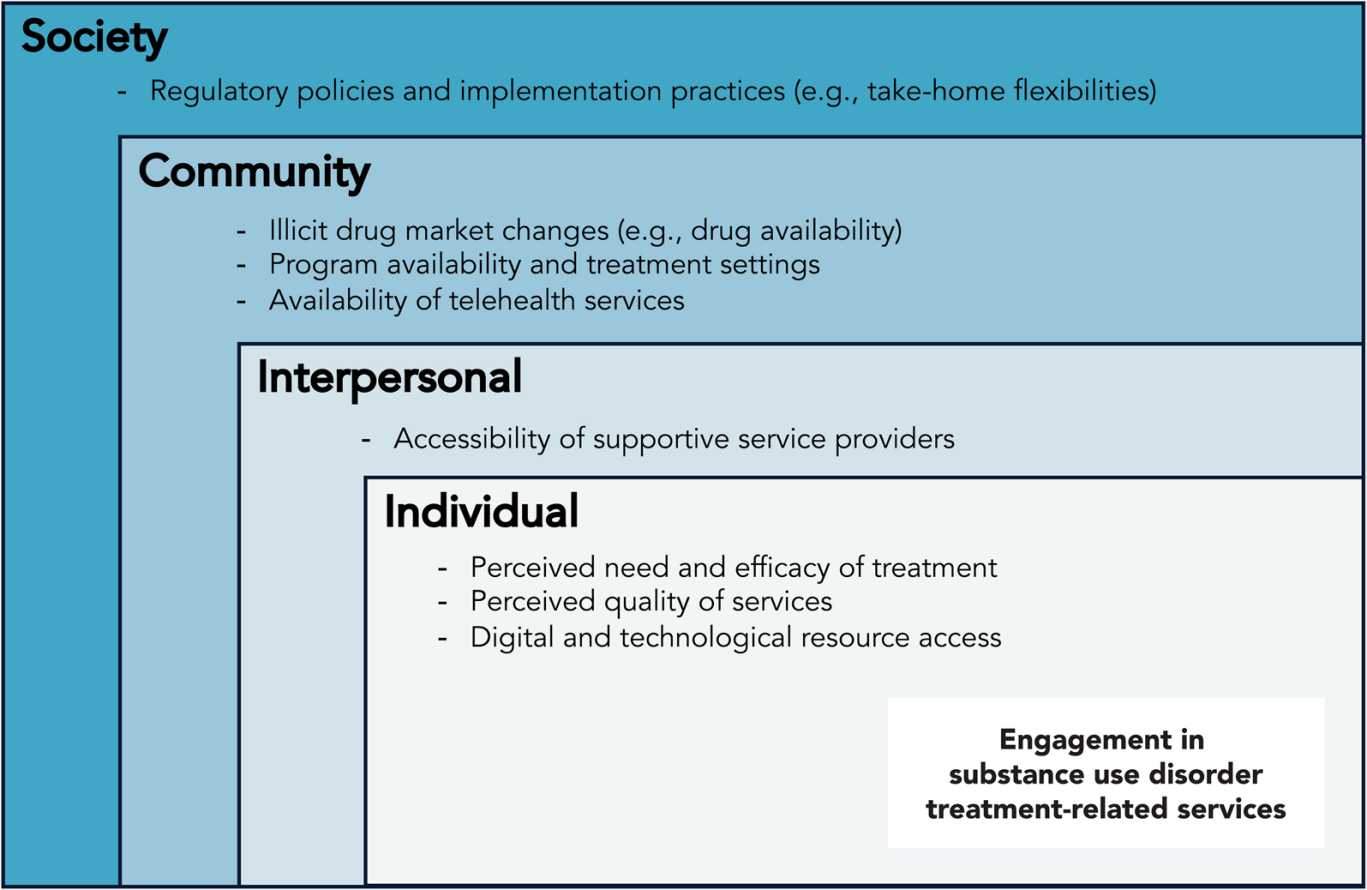
Socioecological model of barriers and facilitators of engagement in substance use disorder treatment-related services during the COVID-19 pandemic

We identified several themes regarding how structural and social changes related to the COVID-19 pandemic influenced individual-level engagement with substance use disorder treatment-related services including MOUD and non-medicinal recovery support services (i.e., individual and group counseling and recovery support groups). Participants described structural and social changes due to the COVID-19 pandemic at the societal-level (e.g., changes in regulatory policies), community-level (e.g., changes in the drug market, service availability and telehealth expansion), and inter-personal level (e.g., increased accessibility of individual service providers), and how these changes acted as barriers and/or facilitators to individual-level engagement in treatment with MOUD and recovery support services. Indeed, some of the structural and social barriers and facilitators at the society- and community-levels also created or exacerbated individual-level barriers to service engagement (e.g., negative attitudes, beliefs, and perceptions). Figure 1 summarizes the salient barriers and facilitators of engagement in MOUD treatment and recovery support services described by participants in the context of the COVID-19 pandemic at each socioecological level.

At the society-level, participants reported substantial changes in the MOUD regulatory environment and related service provision that facilitated engagement in treatment with MOUD (e.g., increased take-home flexibilities) and served as a barrier to engagement in treatment with MOUD (e.g., rescinded take-home flexibilities). At the community-level, participants reported changes in the illicit drug market, such as reduced drug availability which facilitated MOUD uptake. However, increased replacement of heroin with fentanyl, exacerbated individual-level beliefs regarding the perceived lack of efficacy of MOUD against fentanyl. Thus, changes in the illicit drug market also served as a barrier to MOUD uptake. In addition, at the community-level, there was decreased availability and modified service provision of in-person recovery support services, which led to many logistical barriers to engagement and a diminished perceived quality of recovery support services. While community-level telehealth expansion facilitated engagement in recovery support services for some participants, it also introduced its own barriers to recovery support service engagement including a digital divide, technological issues and limited perceived quality of virtual formats. Finally, at the inter-personal level, increased accessibility of individual service providers facilitated participants’ ability to access recovery support. We did not find meaningful differences in these themes by age, sex, or race. The thematic findings are detailed below.

### Society-level changes in the regulatory environment and their impact on individual-level engagement in treatment with MOUD

#### Facilitator: take-home MOUD flexibility policies

Many participants reported no change or temporary increases in access to MOUD during the pandemic, most of which was attributed to temporary take-home flexibilities that required less frequent visits to substance use disorder treatment programs. Attending the substance use disorder treatment program bi-weekly or weekly rather than daily to obtain methadone was “*a big plus*” for participants. However, many participants, including a 55-year-old, non-Hispanic, unspecified race, female participant who re-engaged in a methadone treatment program during the pandemic, clarified that these take-home flexibilities were only temporary:

*So when COVID hit, I was actually getting two weeks at a time. Yeah, because they gave us a COVID week, and then I had the week that I earned to where I could get take-homes. And now it’s harder. It was harder to get your bottles—I mean, it was easier to get your bottles during COVID. Now, they’re back to you have to earn those bottles. And they will take those bottles real quick if you screw up. They don’t just give you bottles anymore. A lot of places don’t. They realize that—and it is. It’s a privilege to get take-home bottles. But I go once a week now. Once a week, me.*

The need to attend treatment facilities less frequently to access MOUD due to service adaptations had additional benefits. Specifically, the increased convenience associated with greater take-home doses of MOUD gave participants more time to engage in other positive health behaviors, including those that participants perceived to be important for their recovery. For example, a 60-year-old, Black male noted:

Participant: *For a while they gave me more take-homes because of my condition and because of the pandemic. See, that—you remember one time everything is like only kind of cleared up because everybody was getting their shots, so then they brought me back more days. But now it’s starting to come back again, another strain of it, so now they’re getting ready to change again.*

Interviewer: *And how has that impacted you and your recovery?*

Participant: *Well I think in a way, it helped me in a way, I got more into my NA [Narcotics Anonymous] books and like that, you know, because I have more free time being by myself.*

Interviewer: *I see. So in your free time you focused more on NA.*

Participant: *Yep.*

#### Barrier: continuation of restrictive pre-pandemic MOUD policies and practices

Despite some participants benefiting from increased take-home methadone flexibilities during the pandemic, many continued to face the same MOUD-related policy barriers that existed before the pandemic. Participants’ ability to take advantage of take-home methadone flexibility policies that were implemented during the pandemic was often countered by other conservative, restrictive, and rigid policies. For instance, several participants discussed how urine drug testing requirements and zero-tolerance policies for ongoing drug use remained a challenge to their access to treatment with methadone. A 60-year-old, Black male participant described how his treatment program took away his take-home methadone bottles due to ongoing drug use:

*Well see they take our urine at least once a month. So they started taking my bottles because the doctor said, “Keep at home now, but—you’ve been home now for, you’ve been home for four months and urine coming up dirty. See I’ve been checking it out, we’ve been checking your urine sample,” she said, “What are you doing? You’re using again.”*

Some participants also described how missing prescribing appointments prohibited them from receiving take-home methadone doses. For instance, a 41-year-old, white male participant who received take-home methadone became comfortable with this new routine during the pandemic but decided to stop engaging in treatment when his take-home flexibility was revoked because he missed an appointment:

Participant: *It was due to the lack of wanting to walk up there. I didn’t want to take my ass up there because they stopped getting the new bottles [take home doses] and I kind of had a temper tantrum and just didn’t feel like going up there and getting them.*

Interviewer: *So, they stopped giving you the take-home bottles?*

Participant: *Yes. They were giving me like six or seven of them, but I missed a day for some reason and they wouldn’t give them back to me. So, I didn’t feel like going up there every day.*

#### Barrier: phase out of take-home MOUD flexibilities

Once take-home flexibility policies phased out during the pandemic for some participants, participants had to resume attending treatment facilities more frequently again to access MOUD, as before the pandemic. Many participants discussed how the increased frequency of required visits to treatment facilities to access MOUD was inconvenient and logistically burdensome. In addition, this barrier was compounded by other community-level structural changes during the COVID-19 pandemic. For example, requirements to frequently attend OTP facilities to access methadone again were logistically challenging for a 54-year-old, Black female participant who relied on public transportation, as public bus routes and schedules were still disrupted by the pandemic:

*Yeah, the buses not running like they normally would. They’re running I’m gonna say a hour and a half different than they used to, and then they done moved bus stops. You’re used to catching it right here, but they don’t stop right here no more. They stop two blocks away now.*

### Community-level changes in the illicit drug market and their impact on individual-level engagement in treatment with MOUD

#### Facilitator: reduced drug availability and a greater perceived need for MOUD

Several participants described decreases in the availability of illicit drugs and lower perceived quality of drugs immediately following the onset of the pandemic. These changes in the illicit drug market had a marked impact on one participant’s MOUD use. Specifically, a 49-year-old, Black female participant was frustrated by a lack of drug availability and was increasingly experiencing withdrawal symptoms, and this experience served as a catalyst for her to initiate methadone for the very first time: “*I got tired of nobody having what I needed at the time of COVID, so I was in here sick, so I said, ‘Let me get on the methadone program,” and that’s what I did.*’ This same participant went on to further describe how initiation of methadone during the pandemic helped her stop using drugs entirely, making her “*look good*” and “*feel good.*”

#### Barrier: perceived lack of efficacy of MOUD against fentanyl

The changes in the drug market associated with the pandemic also exacerbated some participants’ perception that MOUD was no longer an effective treatment option. Specifically, participants described how heroin was mostly replaced by fentanyl during the pandemic, and a couple participants further explained their belief that MOUD is not efficacious against the potency of fentanyl that was available during the pandemic. For example, a 51-year-old, white male participant noted:

*I smoke crack, and I do fentanyl, which used to be heroin, but the heroin disappeared, and now they put pills with fentanyl in them. And I’m on a methadone program, but it doesn’t help with the fentanyl, and so there’s nothing to really curb that sickness when you start feeling bad but more fentanyl, <laughs> so I don’t know what the heck to do with it, but, yeah, that’s my usual routine.*

A 44-year-old, white male participant on methadone also noted, *“I’m already on methadone but fentanyl, you know what I’m saying, it don’t, you know— methadone don’t or the other one don’t block fentanyl. There’s nothing for fentanyl.”*

### Community-level changes in the availability and provision of in-person services and their impact on individual-level engagement in recovery support services

#### Barrier: program closures and service adaptations (e.g., reduced frequency and capacity)

A few participants described how many treatment programs and community-based organizations that provided recovery support services, including counseling and support group meetings, closed down following the onset of the pandemic. For example, a 73-year-old, Black male participant noted, “*they done closed all the places where they had meetings at*.” Many participants also described how some programs continued to provide MOUD during the pandemic, but stopped providing in-person recovery support services, including group counseling and recovery support group meetings. For example, a 54-year-old, white female participant mentioned, “*they don’t let you go to groups now because of the pandemic. There’s no groups.* <*laughs*> *They won’t let you go to groups at all. You come in, you get your dose and your bottles, and you leave.”*

A 52-year-old, Black male participant also highlighted a similar disruption to his daily routine and recovery process:

*Well, with my NA groups, I really—I had grown accustomed to those, because I was going every day. And when they just stopped, I was like, “Okay, well, I can deal with this. I can get past this.” Because I thought it was only going to be for a short time. I thought it was only going to be for a month, maybe two at the most. And it turned out to be a year and a half later.*

Many participants also elaborated on how treatment programs adapted to the pandemic by implementing COVID-19 safety precautions for in-person group counseling sessions and peer recovery support group meetings, such as decreased frequency of meetings, required use of face coverings, and/or decreased capacity limits to enable physical distancing, which disrupted their access to these services. For example, a 55-year-old, white male participant clarified, “*they hardly have the groups anymore. They have them maybe once a month and I used to go there at least a couple times a week.*”

#### Barrier: diminished perceived quality of in-person services

The service adaptations made to in-person group counseling and recovery support group meetings led many participants to express that in-person services had diminished in quality as compared to before the pandemic. Specifically, many participants described how in-person recovery support group meetings during the pandemic did not foster social connection and felt less meaningful as compared to before the pandemic. A 52-year-old, Black male participant noted how COVID-19 related discussions at in-person recovery support group meetings that re-opened during the pandemic contributed to his own anxiety, which led to decreased engagement in these services:

*The groups haven’t really been like they used to be. And to really tell you the truth, I don’t even want to deal with all that right now. I don’t want to deal with all those people right now. Because I don’t want to keep on hearing about the bad parts, about the vaccine, what they think about this new delta virus. And I don’t want to—I’m not trying to get myself worked up about all that.*

Some other participants did not attend in-person recovery support groups altogether because they were uncomfortable being around other people and were worried about contracting COVID-19 during them. A 73-year-old, Black male participant explained how he decreased his engagement in in-person recovery support group meetings to minimize his risk of dying from COVID-19 despite the precautions implemented:

Participant: *I’m not trying to be around a whole lot of people.*

Interviewer: *Yeah. And what was your reason for not wanting to be around people very much?*

Participant: *Because I don’t want to die. Yeah. I don’t want to die.*

Interviewer: *So you were just trying to reduce your COVID risk?*

Participant: *Absolutely. Absolutely. Just like they suggested.*

A couple participants who valued recovery support group meetings before the pandemic directly attributed the diminished access and perceived inferior quality of recovery support group meetings during the pandemic to increased drug use. For instance, a 54-year-old, white female participant noted:

*They [recovery support group meetings] do help. Don’t get me wrong. They help, and they helped a lot. They helped a lot, but you don’t get that anymore, so I guess that’s why I use a lot more now, so...*

### Community-level telehealth expansion and its impact on individual-level engagement in recovery support services

#### Facilitator: availability of virtual recovery support services

Many treatment programs and community-based organizations adapted to the pandemic by implementing virtual recovery support services (e.g., videoconferencing), particularly when they were unable to provide in-person services. Some participants reported that virtual platforms served to maintain access and engagement in recovery support services during the pandemic. For example, a couple of participants noted the convenience of virtual access to peer recovery support group meetings. These participants explained how the virtual meetings enabled them to foster social connections with others with shared lived experience and expand their peer support network beyond their local community. A 69-year-old, Black male participant noted how virtual recovery support group meetings provided him with a higher level of understanding of addiction that he did not have prior to the pandemic:

*I don’t know, basically I kind of enjoyed it, you know? Like I kind of enjoyed it because, you know, it’s basically like you—because the meetings, you can get online with the meetings just anywhere around the world… And then, you know, like it showed me that—you know, that as far as addiction is concerned, it’s not something that’s going on just locally, it’s really affected everybody around the world, basically. And everybody trying to change their life.*

#### Facilitators: access to digital and technological resources

For the participants who were able to virtually access recovery support group meetings, having access to the necessary digital and technological resources was key to facilitating their engagement in the virtual meetings. For one 39-year-old, Black female participant, a structural program like the federal Emergency Broadband Benefit (EBB) program—which provided financial support to low-income households to cover costs for broadband services and certain devices during the pandemic—was pivotal to her ability to access and engage in virtual recovery support group meetings:

Participant: *Now, when COVID hit like having the Internet is mandatory. That’s why thank goodness they came out with the EBB program, you know, emergency broadcast thing for people with low income or no income to get free phones or free Wi-Fi and things like that.*

Interviewer: *I see. You said everything moved online after COVID. Were you able to access those treatments then like groups or meetings?*

Participant: *Yeah. With my [new] phone yes. I was doing NA meetings online and things like that. I was on everything online.*

#### Barrier: digital divide—inadequate digital and technological resources

In contrast, several participants expressed difficulties in accessing virtual recovery support group services due to a lack of required digital and technological resources (i.e., phones with a camera, computers, internet access). A 67-year-old, Black male participant noted: “*They [support group meetings] were available online, but I didn’t take advantage of that, I didn’t have 100 percent access to the internet or a computer.*”

Some participants who did have access to basic digital and technological resources still faced technological issues when attending virtual recovery support group meetings. For instance, a 52-year-old, Black male participant explained:

*I did one virtual meeting. It wasn’t to my liking. I didn’t care for it too much. Well, the virtual meetings, the contact wasn’t what I was looking for. And then my phone kept on dropping it, dropping the meeting. And it just got frustrating.*

#### Barrier: poor perceived quality of virtual services

In addition to being frustrated by the technological issues experienced during virtual recovery support group meetings, some participants highlighted that they felt a lack of social connection with others and experienced competing cognitive demands that made it difficult to pay attention and remain actively involved. For example, a 31-year-old, white female participant noted feeling “*less motivated to get involved*” virtually:

*It’s been different. You know, doing it on Zoom or whatever…I guess it’s not as connected. It’s just—I don’t know how to explain it. I don’t know. You can just get left out, if you don’t put yourself in there, because it’s all on the computer.*

### Inter-personal level changes in accessibility of individual service providers and their impact on individual-level engagement in recovery support services

#### Facilitator: increased access to individual service providers

Several participants described how long-standing supportive relationships with their counselors at substance use disorder treatment programs provided stability in their recovery process at the time of the pandemic. Given structural changes in substance use disorder treatment programs that decreased contact with clients, counselors adapted to maintain engagement with clients to facilitate participants’ access to support. Some participants particularly valued these adaptations, especially when their usual in-person recovery support group meetings were not available to them. For example, a 55-year-old, non-Hispanic, unspecified race, female participant who did not like the quality of virtual recovery support group meetings during the pandemic reported relying more on her methadone counselor, as her counselor made herself available to her whenever she needed, even outside of structured clinic hours: “*I have my counselor at my [methadone] program. I actually have her cell phone number so I can call her at home, because she doesn’t want anything to happen to me again, to where I shut down.”*

In addition to outpatient counselors, a 54-year-old, Black female participant with limited access to technology described a positive relationship with her peer mentor (i.e., 12-step sponsor) who went beyond their normal practice to provide individual- and group-based social and recovery support services to their clients during the pandemic:

*Yeah, I don’t have that phone [with a video camera], so what I had to do was stay in touch more with my sponsor, right, and then my sponsor started having small meetings, all-women, at her house or at her best girlfriend house. She was doing that like once a month. She still do it. We still do that once a month, right, just to try to help us stay connected, but I talk to my sponsor every other day, sometimes every day, yeah, and she keeps a close eye on me, which I need, because sometimes I fall short, but I pick myself back up, and I keep going. I don’t stay stuck in that, so I’m just trying to get back to where I was, because I had five years clean, and I had messed-up, but I went right in the meeting and told them all. I told them. I say “I shouldn’t be up here secretary-ing the meeting or sharing.” I say “I messed-up last night, and I used, and I don’t know why.” Well, I do know why. I do. I do know why. Because I was putting all my focus and energy into something else instead of into my meetings and groups like I was, okay?*

## Discussion

This study explored how structural and social changes due to the COVID-19 pandemic impacted individual-level experiences with substance use disorder treatment-related services among a community-based sample of people with a recent history of injection drug use in Baltimore, Maryland. In this sample, the COVID-19 pandemic led to many structural and social changes across various socioecological levels that influenced participants’ engagement with substance use disorder treatment-related services. At the society-level, participants reported that the take-home methadone flexibility policies temporarily helped to engage in MOUD use, but these flexibilities were countered by rigid policies that required abstinence from drug use and punctual in-person attendance for regularly scheduled visits at OTPs to receive take-home doses. At the community-level, the pandemic significantly disrupted participants’ access to and engagement in recovery support services as there were program closures and mixed access to and engagement in virtual platforms for these services. Although telehealth expansion helped many participants maintain access to recovery support services— particularly recovery support group meetings—during the pandemic, participants also perceived a lower quality of both virtual and in-person recovery support services during the pandemic. At the inter-personal level, increased accessibility of supportive counselors and 12-step sponsors (outside of normal practice) was key to facilitating recovery support during the pandemic.

Society-level changes in US regulatory policies for methadone provision in response to the pandemic were key to ensuring access to this life-saving medication. Many participants described taking advantage of take-home methadone flexibilities and how weekly trips to their OTP were preferable and more convenient than making near-daily visits. This value for take-home methadone flexibilities and reduced OTP visits is consistent with what has been reported in qualitative studies of other populations of people who use drugs in the US [92–95]. Policy makers and providers have also reported positive experiences with relaxing restrictions on take-home MOUD during the pandemic and have endorsed shifting towards “low threshold” policies and person-centered care [96–98]. However, stigma by some policymakers and providers continues to be a challenge to the adoption and implementation of such relaxed policies [98, 99]. Notably, our data highlight how some programs scaled back take-home flexibilities and that participants’ qualifications for receiving take-home doses were dynamic depending on their level of ongoing drug use and attendance to their OTP. Revoking methadone take-home flexibilities led to dissatisfaction with participants’ treatment experience and had a negative impact on some participants’ recovery process, such as for the one participant who completely disengaged from treatment with methadone because he no longer qualified for take-home flexibilities. This is consistent with quantitative evidence demonstrating that dissatisfaction with the MOUD treatment experience is associated with treatment discontinuation [100]. Overall, these data add to the growing evidence base in favor of continuing take-home methadone flexibilities [51].

Our findings also demonstrate how community-level shifts in the illicit drug market can heterogeneously influence individual-level engagement in treatment with MOUD. Some participants described how the COVID-19 pandemic decreased drug availability and quality during the pandemic; in a phone-based survey of ALIVE participants during the initial months of the pandemic, we also found that 19% of current and former PWID who were actively using drugs reported problems findings drugs in the prior 2 weeks [66]. Community-level changes in drug availability, quality, and price during the pandemic have also been reported by people who use drugs in other settings [72, 101–103]; however, there is limited evidence as to how these community-level shifts in the illicit drug market impacted individual-level experiences with MOUD use. One participant, who was unable to find drugs during the early pandemic, initiated methadone to help address their opioid dependence and withdrawal symptoms. While this finding should be cautiously interpreted given it was only observed among one participant in our sample, it is a salient example for why relaxed policies and environments are needed to facilitate access to MOUD initiation. It is also notable that while community-level shifts in the illicit drug market were already occurring before the pandemic in Baltimore, with increased adulteration of illicit drugs with fentanyl and fentanyl analogs and an overall increased availability and use of fentanyl [104–108], some participants noted they could only find fentanyl during the pandemic. This exacerbated individual attitudes regarding perceived lack of efficacy of MOUD against the potency of fentanyl during the pandemic, highlighting how structural changes at the community level can interact with individual-level perceptions and beliefs to influence engagement in health services. This negative perception of MOUD has also been reported by other populations of people who use drugs before and during the pandemic [109]. Providers should collaborate with clients to optimize MOUD doses to match the client’s need, which may require higher doses to achieve a therapeutic response among clients using fentanyl [110].

Support from peers and allies is a guiding principle for achieving and maintaining recovery. Indeed, social support has been associated with a myriad of health outcomes among PWID, including mental and emotional well-being [69, 111], injection cessation [112, 113], and a reduced risk of overdose [114]. We previously reported that the early COVID-19 pandemic period led to perceived stress from social isolation and a perceived increase in loneliness among PWID in Baltimore [69]. PWID in other settings have also reported decreased social support and increased feelings of social isolation because of the pandemic [62, 115]. Recovery support services have historically been shown to be an important source of social support for people who use drugs [33, 116, 117], and our data demonstrate that the pandemic caused major community-level disruptions in access to recovery support services, particularly recovery support group meetings. The structural barriers to engagement in recovery support group meetings during the pandemic included program closures and suspension of in-person recovery support group meetings, which is consistent with what has been reported by PWID and service providers elsewhere [62, 101]. Many participants reported that attending recovery support group meetings was important to their recovery process, and the lack of access to these in-person meetings during the pandemic disrupted their daily routine. Some participants reported that the lack of access to in-person recovery support group meetings during the pandemic led to an increase in their drug use. Strategies are needed to ensure continuity in access to recovery support services during times of social disruption.

Indeed, many substance use disorder treatment programs and community-based organizations adapted to the pandemic by offering in-person recovery support group meetings with COVID-19 risk mitigation procedures (e.g., limited capacity) as well as implementing virtual recovery support group meetings (i.e., telehealth expansion). These adaptations generally helped to maintain access to recovery support group meetings during a time of social disruption, with some participants appreciating the ease of access of virtual platforms. However, participants who were able to attend recovery support group meetings during the pandemic also noted that there was a loss of interpersonal and social connection during the in-person and virtual meetings. This perception of reduced efficacy and quality (i.e., limited acceptability) of in-person and virtual recovery support group meetings during the pandemic has also been reported in other populations and settings [118, 119]. Research is needed on how to improve the client experience of both the in-person and virtual modes of recovery support group meetings, including the implementation of rapport-building strategies on virtual platforms. It is also notable that some participants had difficulties making the transition from in-person to virtual group meetings owing to resource and technological barriers. Structural programs, such as the EBB program, that provide the resources needed to attend virtual recovery support services to priority populations may be critical for addressing the digital divide for virtual recovery support services. Equitable access to both in-person and virtual platforms for recovery support services may help to improve overall engagement in these services among PWID.

Methadone and behavioral health counselors are also critical to creating a supportive treatment environment, and peer recovery coaches and 12-step sponsors can also be key sources of social support [33, 120–122]. Participants reported that some programs stopped providing in-person counseling services and that they were unable to meet with their 12-step sponsor during the pandemic. However, we also found that some counselors and sponsors individually adapted to the pandemic to increase their accessibility to their clients (e.g., providing their personal cell-phone numbers and offering virtual group sessions) and continue to provide recovery support. Some participants really valued the support efforts made by the providers with whom they had previously developed a meaningful relationship. Although prior research suggests service providers including methadone counselors at OTPs can be a source of enacted and anticipated stigma for their clients [123–126], these data suggest supportive providers can also have positive influences on their clients. Other studies have reported operational challenges in MOUD provision including staffing reductions and staff turnover at OTPs and office-based treatment programs during the pandemic [127, 128], which may be disruptive to maintaining supportive relationships between clients and their providers as well as the clients’ engagement in substance use disorder treatment-related services. Substance use disorder treatment programs need the resources to facilitate the retention of trained service providers who may have long-standing relationships with their clients.

This study has limitations that merit consideration. While it is a strength that the study covered an extended period following the onset of the COVID-19 pandemic, this may have impacted participants’ recall of their experiences with and perceptions of substance use disorder treatment-related services before and during the pandemic. It is also a strength that these data represent the perspectives of current and former PWID who were in and out of care for their substance use disorder; however, we used a convenience sampling approach and conducted the interviews exclusively by telephone, which limited the sample to those who had access to a telephone. Accordingly, we were unable to capture the experiences of people with a history of injection drug use who do not have access to a telephone. Given the unique urban context of Baltimore with relatively many healthcare resources, it is likely that barriers and facilitators of substance use disorder treatment-related services may differ in rural settings in the US [129]. We also did not find meaningful differences by age, sex, or race and ethnicity in our sample; however, our sample was skewed toward an older age range. It is possible that PWID of different ages may face unique barriers and facilitators of substance use disorder treatment-related service utilization. Finally, it should be noted that telemedicine for buprenorphine was not reported by this study sample; thus, we were unable to characterize experiences accessing buprenorphine via telemedicine. It is possible that telemedicine may not have been a salient facilitator of buprenorphine use for this population.

## Conclusions

This study highlights how structural and social changes across multiple socioecological levels can create new barriers and facilitators of engagement in substance use disorder treatment-related services for people with a recent history of injection drug use. For instance, the regulatory changes pertaining to flexible methadone provision were helpful in promoting engagement in MOUD use and have potential to further expand MOUD uptake. However, our data suggest the benefits of these take-home flexibilities were short-lived for some participants and were not maximized for others, due to pre-existing conservative and rigid policies and practices regarding methadone provision. Additionally, there were significant disruptions in access to, engagement in, and perceived quality of recovery support services during the COVID-19 pandemic, despite community-level adaptations such as the expansion of telehealth. The society- and community-level changes described in this study had downstream negative impacts on the treatment engagement and recovery process for some PWID, highlighting how the structural and social consequences of the COVID-19 pandemic have impacted the risk environment. These data provide important lessons applicable to improving substance use disorder treat-related service delivery in everyday practice. Multilevel strategies and approaches are needed to enable access to and engagement in comprehensive and high-quality substance use disorder treatment-related services among priority populations, especially during future social disruptions.

### Supplementary Information

**Additional file 1.** CHANGES interview guide.

**Additional file 2.** Consolidated criteria for Reporting Qualitative research (COREQ) checklist.

## Data Availability

Data are not publicly available due to the sensitive nature of the data. Data requests can be submitted to the principal investigator (bgenberg@jhu.edu).

## Abbreviations

AIDS: Acquired immune deficiency syndrome
ALIVE: AIDS Linked to the IntraVenous Experience
COVID-19: Coronavirus disease 2019
COREQ: Consolidated Criteria for Reporting Qualitative research
CHANGES: Covid Health and Network Group Experience Study
DEA: Drug Enforcement Administration
EBB: Emergency Broadband Benefit
HCV: Hepatitis C virus
HIV: Human immunodeficiency virus
MOUD: Medications for opioid use disorder
NA: Narcotics Anonymous
OTP: Opioid Treatment Program
PWID: People who inject drugs
SAMHSA: Substance Abuse and Mental Health Services Administration
US: United States
