# Supplementary material for: Structural and social changes due to the COVID-19 pandemic and their impact on engagement in substance use disorder treatment services: a qualitative study among people with a recent history of injection drug use in Baltimore, Maryland
Source: Harm Reduct J. 2024 May 8;21:91. doi: 10.1186/s12954-024-01008-8 (PMC11077846; doi:10.1186/s12954-024-01008-8)
Supplement: Supplementary file 1 — Additional file 1. CHANGES interview guide. [file 12954_2024_1008_MOESM1_ESM.pdf]

THE ALIVE STUDY

COVID-19 NETWORK QUALITATIVE SURVEY

GENERAL INFORMATION

SUBJECT ID: _____ _____ _____ _____ _____

FORM VERSION: 1.0

DATE OF INTERVIEW: _____ _____ / _____ _____ / _____ _____

INTERVIEWER'S INITIALS: _____ _____ _____

TIME INTERVIEW BEGAN: _____ _____ : _____ _____

BACKGROUND: The goal of this discussion is to understand changes to the participant's lifestyle and network since the start of the COVID-19 pandemic. We would like to get a sense of the factors mediating changes to their social network, drug network, and drug use behaviors to inform the questions we ask in the quantitative survey.

READ TO THE PARTICIPANT:

The purpose of this interview is to understand how the COVID-19 pandemic has impacted you, your relationships with others, and your drug use behaviors. I will ask you a series of open-ended questions that you should answer as honestly as possible. I'll be taking notes on what you say, and I'd like to record the interview so that I can make sure that I don't miss anything you say. Your name will not be recorded. The recording will not be shared with anyone outside of the study team and will be destroyed after the study is complete.

1. "Catching up": *Broad overview of how the participant's lifestyle and network has changed as a result of COVID*

a) I'd like to start by asking you to tell me about how your day-to-day life has changed in the past year, since the start of the COVID-19 pandemic (March 2020).
b) **NOTE FOR INTERVIEWER:** Ask these follow-up questions if the participant does not address them in their initial answer.
    i. How has your routine changed since the start of the COVID-19 pandemic? How are things different now from what they were like at the beginning of the pandemic?
    ii. Has your source(s) of income changed recently or since the start of the COVID-19 pandemic? If so, how has it changed?
    iii. Has your primary place of residence changed recently or since the start of the COVID-19 pandemic? If so, how has it changed?
    iv. Have your family commitments or responsibilities changed since the start of the COVID-19 pandemic (e.g. taking on a caretaker role)?
    v. Has your health changed? If so, how has it changed?
    vi. Has your experience with medical services (e.g. HIV care) or mental health services changed since the start of the COVID-19 pandemic? Have you faced any difficulties accessing these services?
c) Tell me about the people in your life you spend the most time with. Who are they, how long have you known them, and what do you spend time doing together?
d) Now, think back to the time before COVID and who you spent time with and were close to then. Is there anyone that you were close with before COVID that you are no longer close with now?
    i. If so, what happened that changed the nature of your relationship?
e) Is there anyone you have grown close to in the past year who you were not close to before COVID?
    i. If so, how and when did you meet them or get to know them better?
f) Have you experienced any other major life events since the start of the COVID-19 pandemic that have changed your routine and/or your relationships with others that you have not already mentioned?

2. Drug use behaviors: *Overview of how participant's relationship with drugs has changed since the start of the COVID-19 pandemic*

a) Now I'd like you to tell me about how your drug use has changed in the past year, since the start of the COVID-19 pandemic (March 2020).
b) **NOTE FOR INTERVIEWER:** Ask these follow-up questions if the participant does not address them in their initial answer.
    i. Have you started or stopped using any drugs in the past year? If so, tell me a bit about the circumstances surrounding that change.

i. **NOTE FOR INTERVIEWER:** The goal is to get at the "why" and "how" here without explicitly asking it.

ii. Has the frequency with which you use drugs changed since the start of the COVID-19 pandemic? Are there any drugs that you are using more or less frequently than before?

iii. Have the methods by which you take drugs changed in the past year? If you started or stopped using any methods of taking drugs (like injecting or smoking), tell me a bit about the circumstances surrounding that change.

iv. Did you quit using drugs altogether at any point? If so, when and what prompted this?

c) Now, I'd like you to think about the people you were using drugs with before the start of the COVID-19 pandemic. Is there anyone that you were using with prior to COVID-19 that you no longer use with now?

  i. If so, what happened to change this?

d) Is there anyone that you are using with now that you were not using with prior to COVID-19?

  i. If so, how and when did you meet them and/or start using with them?

e) Before the COVID-19 pandemic, were you enrolled in drug or alcohol treatment, attending any support groups, or accessing any other drug services (e.g. needle exchanges or syringe services programs)? If so, tell me about how your experiences with these services have changed in the past year.

f) **NOTE FOR INTERVIEWER:** Ask these follow-up questions if the participant does not address them in their initial answer.

  i. Has the frequency with which you are accessing these services changed?

  ii. Have you faced any difficulties accessing these services?

g) Have you started accessing drug or alcohol treatment, support groups, or any other drug services since the start of the COVID-19 pandemic?

  i. If so, tell me a bit about your experiences with these services and what prompted you to access them.

h) Has your relationship with drugs changed since the start of the COVID-19 pandemic in any other ways that you have not already mentioned?
